# Predicting success in the worldwide start-up network

**DOI:** 10.1038/s41598-019-57209-w

**Published:** 2020-01-15

**Authors:** Moreno Bonaventura, Valerio Ciotti, Pietro Panzarasa, Silvia Liverani, Lucas Lacasa, Vito Latora

**Affiliations:** 1 School of Mathematical Sciences, Queen Mary University of London, Mile End Road, E14NS London, UK; 2 School of Business and Management, Queen Mary University of London, Mile End Road, E14NS London, UK; 3 The Alan Turing Institute, The British Library, NW12DB London, UK; 4 Dipartimento di Fisica e Astronomia, Università di Catania and INFN, 95123 Catania, Italy; 5 Complexity Science Hub Vienna (CSHV), Vienna, Austria

**Keywords:** Complex networks, Scientific data

## Abstract

By drawing on large-scale online data we are able to construct and analyze the time-varying worldwide network of professional relationships among start-ups. The nodes of this network represent companies, while the links model the flow of employees and the associated transfer of know-how across companies. We use network centrality measures to assess, at an early stage, the likelihood of the long-term positive economic performance of a start-up. We find that the start-up network has predictive power and that by using network centrality we can provide valuable recommendations, sometimes doubling the current state of the art performance of venture capital funds. Our network-based approach supports the theory that the position of a start-up within its ecosystem is relevant for its future success, while at the same time it offers an effective complement to the labour-intensive screening processes of venture capital firms. Our results can also enable policy-makers and entrepreneurs to conduct a more objective assessment of the long-term potentials of innovation ecosystems, and to target their interventions accordingly.

## Introduction

Recent years have witnessed an unprecedented growth of interest in start-up companies. Policy-makers have been keen to sustain young entrepreneurs’ innovative efforts with a view to injecting new driving forces into the economy and fostering job creation and technological advancements^1–4^. Investors have been lured by the opportunity of disproportionally high returns typically associated with radical new developments and technological discontinuities. Large corporations have relied on various forms of external collaborations with newly established firms to outsource innovation processes and stay abreast of technological breakthroughs^5^. Undoubtedly, knowledge-intensive ventures such as start-ups can have a large positive impact on the economy and society. Yet they typically suffer from a liability of newness^6^, and cannot avoid the uncertainties and sunk costs resulting from disruptive product developments, uncharted markets and rapidly changing technological regimes^7^. For these reasons, their long-term benefits are inherently difficult to predict, and their economic net present value cannot be unambiguously assessed^8^.

Indeed traditional models of business evaluation, based on historical trends of data (e.g., on sales, production capacity, internal growth, and markets size) are mostly inapplicable to start-ups, chiefly because their limited history does not provide sufficient data. Venture capitalists and private investors often evaluate start-ups primarily based on the qualifications and dexterity of the entrepreneurs, on their potential to create new markets or niches and to unleash the “gales of creative destruction”^9^. The process of screening and evaluating companies in their early stages is therefore a subjective and labor-intensive task, and is inevitably fraught with biases and uncertainty.

To overcome these limitations, we propose a novel and data-driven framework for assessing the long-term economic potential of newly established start-ups. Our study draws upon the construction and analysis of the worldwide network of professional relationships among start-ups. Such network provides the backbone and the channels through which knowledge can be gained, transferred, shared, and recombined. For instance, skilled employees moving across firms in search of novel opportunities can bring with them know-how on cutting-edge technologies; advisors who gained experience in one firm can help identify the most effective strategies in another, whilst well connected investors, lenders and board members can rely on the knowledge gained in one firm to tap business and funding opportunities in another.

Previous work has investigated how knowledge transfer impacts upon the performance of start-ups; yet information flows have been simply inferred mainly through data on patents^10^, interorganizational collaborations^11^, co-location of firms and their proximity to universities^12^. Other studies have analyzed social networks (e.g., inventor collaboration networks, interlocking directorates) to unveil the microscopic level of interactions among individuals; yet their scope has been limited mostly to specific industries or small geographic areas, and to a fairly small observation period^11,13,14^. Owing to lack of data, what still remains to be studied is the global network underpinning knowledge exchange in the worldwide innovation ecosystem. Equally, the competitive advantage of differential information-rich network positions and their role in opening up, expediting, or obstructing pathways to firms’ long-term success have been left largely unexplored.

## The World-Wide Network of Start-Ups

Here we study the complex time-varying network^15,16^ of interactions among all start-ups in the worldwide innovation ecosystem over a period of 26 years (1990–2015). To this end, we collected all data on firms and people (i.e., founders, employees, advisors, investors, and board members) available from the www.crunchbase.com website. Drawing on the data, we first constructed a bipartite graph in which people are connected to start-ups according to their professional role. We then obtained the projected one-mode time-varying graph in which start-ups are the nodes and two companies are connected when they share at least one individual that plays or has played a professional role in both companies (see Supplementary Information (SI) for details). At the micro scale, employees working in a company can perceive the intrinsic value of new appealing opportunities and switch companies accordingly. This mobility creates an information and intel flow between companies, such that those receiving employees increase their fitness by capitalizing on the know-how the employees bring with themselves. Such microscopic dynamics is thus captured and modelled here by the creation of new edges at the level of the network of start-ups. As a consequence, companies which are perceived at the micro scale as appealing opportunities by mobile employees will likely boost their connectivity and therefore will acquire a more central position in the overall time-varying network. Note that ideas revolving about the hypothesis that the position of a start-up within its ecosystem is relevant for its future success have been previously discussed by some authors^17,18^ and more recently formalised by Sorkin^19^. For simplicity, here we assume edges between companies to be undirected (which reflects more knowledge sharing than transfer) as while the movement of an employee from company A to company B certainly boosts the know-how of B and under our approach should thus increase its centrality, it does not necessarily decrease the know-how and centrality of A. Similarly, we assume memory to be present and thus keep all edges in the network, i.e. edges are not deleted over time as know-how is not necessarily destroyed (see SI Section 5.4 for details).

**Figure 1:**
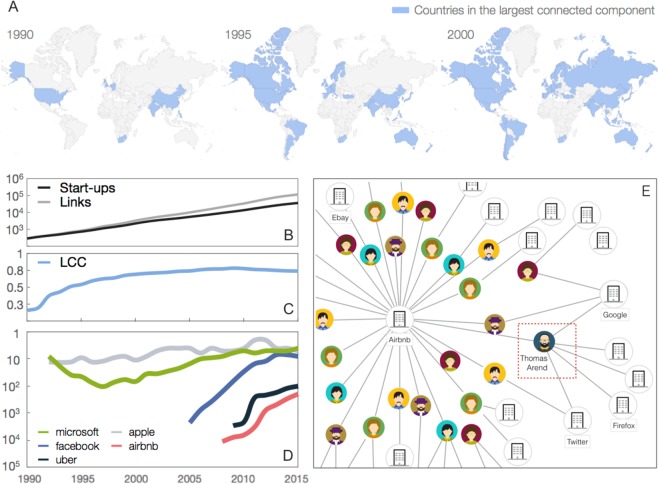
The time-varying network of professional relationships among start-ups. (**A**) Countries that, over time, joined the largest connected component (LCC) of the worldwide start-up (WWS) network are highlighted in blue. (**B**) Evolution over time of the number of firms and links in the WWS network. (**C**) Evolution over time of the fraction of nodes in the LCC. (**D**) Evolution over time of the closeness centrality rank of five popular firms. (**E**) Airbnb’s ego-centered network (Icon faces are by https://icon-library.net/icon/human-face-icon-2.html/CC0 Public Domain Licence).

The resulting time-varying *World Wide Start-up* (WWS) network comprises 41,830 companies distributed across 117 countries around the globe, and 135,099 links among them (see SI Figs. S1 and S2). Figure 1A highlights the countries in which start-ups have joined, over time, the largest connected component of the network^15,16^. Figure 1B indicates that the number of nodes and links in the WWS network has grown exponentially over the last 26 years. In the same period, various communities of start-ups around the globe have joined together to form the largest connected component including about 80% of the nodes of the network (Fig. 1C). Currently, an average of 4,74 “degrees of separation” between any two companies characterizes the WWS network.

At the micro scale, Fig. 1E shows a snapshot of the network of interactions between Airbnb and other companies based on shared individuals. As an illustration, in 2013 Airbnb hired Mr Thomas Arend (highlighted in the red square), who had previously acted as a senior product manager in Google, as an international product leader in Twitter, and as a product manager in Mozilla. As previously pointed out, the professional network thus reveals the potential flow of knowledge between Airbnb and the three other companies in which Mr Arend had played a role. Moreover, as new links were forged over time, the topological distances from Airbnb to all other firms in the WWS network were reduced, which in turn enabled Airbnb to gain new knowledge and tap business opportunities beyond its immediate local neighborhood.

The mechanistic interpretation of employees’ mobility inducing link creation discussed above and illustrated in Fig. 1E suggests that the potential exposure to knowledge of a start-up in the WWS network, and its subsequent likelihood to excel in the future, should be well captured by its network centrality over time. To test this hypothesis we have considered different measures of node centrality^20^. For parsimony here we focus on the results obtained using *closeness centrality* as it assesses the centrality of a node in the network from its average distance from all the other nodes, although similar results has also been found using some other centrality measures, such as betweenness or degree (see SI). In each month of the observation period, we ranked companies according to their values of closeness centrality (i.e., top nodes are firms with the highest closeness). Figure 1D is an example of the large variety of observed trajectories as companies moved towards higher or lower ranks, i.e., they obtained a larger or smaller proximity to all other companies in the network. Notice that Apple has always been in the Top 10 firms over the entire period, while Microsoft exhibited an initial decline followed by a constant rise towards the central region of the network. The trajectories of formerly younger start-ups, such as Facebook, Airbnb, and Uber, are instead characterized by an abrupt and swift move to the highest positions of the ranking soon after their foundation, possibly as a result of the boost in activity that has characterized the venture capital industry in recent years.

## Early-Stage Prediction of High Performance

To investigate the interplay between the position of a given firm in the WWS network and its long-term economic performance, from www.crunchbase.com we collected additional data on funding rounds, acquisitions, and initial public offerings (IPOs). For each month $t$, we obtained the list of $N(t)$ firms, ranked in terms of closeness, that can be classified as “open deals” for investors, namely: (i) they have not yet received funding; (ii) they have not yet been acquired; and (iii) they have not yet been listed in the stock exchange market (see SI Fig. S3). As an example, the company WhatsApp, which ranked 1,060^th^ in June 2009 in the full list, occupied the 15^th^ position in the open-deals list in the same month. Notice that, by assessing a firm’s network position prior to any financial acquisition or IPO, our analysis is not subject to possible biases arising from the effects that the capital market might have upon the firm’s expected performance. Furthermore, predicting the long-term economic performance of firms in the open-deal list is arguably a challenging task, as illustrated by the fact that the average success of venture capital funds focused on early-stage investments in similar open deals is only around 10–15% (see SI Section 4.2 for a table on average success of venture capital funds focusing in comparatively similar early-stage companies). Over the range of 26 years of the dataset, a total of 5305 different start-ups were identified as open-deals.

Our recommendation method is based on the hypothesis that start-ups with higher values of closeness centrality at an early stage are more likely to show signs of positive long-term economic performance. Accordingly, we counted the total number $m(t)$ of firms inside the open-deal list that, within a time window $\Delta t=7$ years starting at month $t$, succeeded in securing at least one of the following positive outcomes: (i) they took over one or more firms; (ii) they were acquired by one or more firms; or (iii) they underwent an IPO. To assess the accuracy of our recommendation method in early identifying successful companies, we checked how many of the Top $n=20$ companies in the closeness-based ranking of open-deals obtained a positive outcome (see SI Fig. S4).

**Figure 2:**
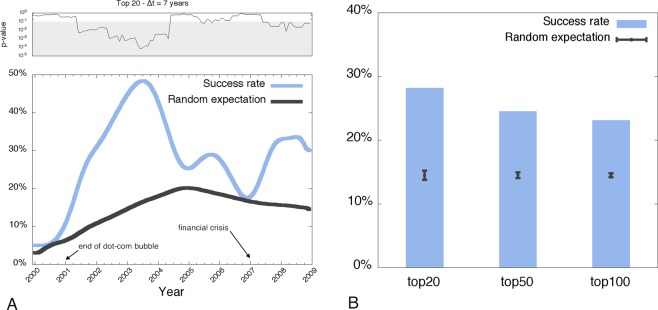
Closeness-based ranking of open-deals and predicting long-term success. (**A**) The performance of our recommendation method in predicting companies’ success on a monthly basis compared to the expected performance of a null model (random ordering of companies). The top panel reports the probability ($p$-value) of obtaining, by chance, a success rate larger than the one observed in the corresponding month. The gray-shaded region indicates the time periods where the prediction is statistically significant (*p*-value < 0.05). (**B**) The overall performance of our method over the entire period of observation based on the Top 20, 50 and 100 firms with the highest closeness centrality. The black error bars indicate the expected success rates and standard deviations in the case of random ordering of companies. Interestingly, results of this null model are comparable to the expected success of those venture capital funds whose portfolio focus on early-stage companies similar to those considered in our open deal list (see Section S4.2 for details), and are about twice as low as our results based on network centrality.

Figure 2A reports the “success rate” *S* (blue curve) of the recommendation method, defined as $S(t)=m(t)/n$, where $m(t)$ is the number of firms with a positive outcome included in the Top $n=20$ firms, and ${S}^{{\rm{rand}}}(t)=M(t)/N(t)$ (black curve) is the success rate expected in the case of random ordering of companies, i.e. the expected success of a null model of random sampling without replacement which complies with a hypergeometric distribution (see SI Section 4). The success rate of our simple heuristic is systematically above the one found with the null model. The $p$-value in the top panel of Fig. 2A measures the probability of obtaining, by chance, a success rate larger than $S(t)$, with low values of $p$ (highlighted regions) indicating the time periods where the prediction is statistically significant (*p*-value < 0.05). From mid 2001 to mid 2004, the success rate of our recommendation method (blue curve) is remarkably larger than the one based on random expectations (black curve), and the $p$-value is always smaller than 0.01. $S(t)$ exhibits an exceptional peak of 50% in June 2003 (*p*-value = 0.0001). From 2004 to 2007, the blue curve decreases, reaching a local minimum at a time when a global financial crisis was triggered by the US housing bubble. In this period (as well as during the collapse of the dot-com bubble in 1999–2001), even though the success rate still exceeds random expectations, the high $p$-values indicate that the predictions are not statistically significant. Finally, after mid 2007, the performance of the prediction increases, and it stabilizes around 35% (*p*-value = 0.01). For completeness, SI Fig. S5 reports results based on different lengths of the recommendation list and on different time windows. Why the performance of our network-based recommendation method seems to depend on the business cycle and on the level of external financial market stress is an important open problem and should be studied in more depth.

In Fig. 2B, we characterize the overall performance of the recommendation method over the entire period of observation. Results indicate that about 30% of the firms appearing in the Top 20 in any month from 2000 to 2009 have indeed achieved a positive economic outcome within 7 years since the time of our recommendation. The black error bars indicate the expected success rates and standard deviations in the case of random ordering of companies ($p$-values in this case are all below 10^−5^). Interestingly, the random null model provides an expected success rate which is indeed comparable to the actual performance that private investors focusing on early-stage start-ups as those considered in our prediction (e.g. accelerators and incubators such as 500 Startups, Y Combinator, Techstars and Wayra, whose target companies comply with our definition of open-deal list) reach through costly and labour-intensive screening processes (see SI Section 4.2 for details), while the performance of our recommendation method is considerably superior.

We further checked the robustness of our methodology by replicating the analysis based on the Top 50 and Top 100 (reported in Fig. 2B), for two additional time windows $\Delta t=6$ and $\Delta t=8$ years (see SI Fig. S6) and an alternative method of aggregation of the success rate across the entire observation period (see SI Fig. S7). We also controlled for different confounding factors such as start-up size, geographical location or structural role of venture capital funds in the start-up network, finding that our conclusions hold (see SI Section 5).

Finally, notice that the method presented here only provides a simple heuristic recommendation, i.e. it does not quantify the probability of *each* start-up in the open-deal list to show economic success in the future. In SI Section 6 we further studied this possibility by using a suite of logistic regression methods to *predict* success of each and every start-up in the open-deal list. We indeed found that a snapshot of the closeness centrality ranking of a given start-up could predict its future economic outcome (F1 score = 0.6), in qualitative agreement with findings in Fig. 2.

## Implications

As lack of data and subjective biases inevitably impede a proper and rigorous evaluation of risky and newly established innovative activities, our study has indicated that the network of professional relationships among start-ups can unlock the long-term potential of risky ventures whose economic net present value would otherwise be difficult to measure. Our recommendation method can help stakeholders devise and fine-tune a number of effective strategies, simply based on the underlying network. Employees, business consultants, board members, bankers and lenders can identify the opportunities with the highest long-term economic potential. Individual and institutional investors can discern financial deals and build appropriate portfolios that most suit their investment preferences. Entrepreneurs can hone their networking prowess and strategies for sustaining professional inter-firm partnering and securing a winning streak over the long run. Finally, governmental bodies and policy-makers can concentrate their attention and efforts on the economic activities and geographic areas with the most promising value-generating potential (e.g., activities with the capacity of job creation, youth employment and skill development, educational and technological enhancement) for both the national and local communities. Sociological and economic research has vastly investigated the impact of knowledge spillovers^21^, involvement in inter-firm alliances^22^ and network position^23^ on firms’ performance, innovation capacity, propensity to collaborate, and growth rates. Yet, whether the centrality in the professional network of newly established knowledge-intensive firms can help predict their long-term economic success has largely remained a moot question. Our work is the first attempt to pave the way in this direction, and represents a contribution, from a different angle, to the ongoing discussion on the science of success^24^, complementing recent findings in different fields such as science^25–27^ and arts^28,29^. Finally, let us note that this work is intended to elucidate the role that network mechanisms might play in sustaining success, rather than to provide more sophisticated yet non-interpretable prediction algorithms. More work should be carried out to fully investigate network-based predictability of economic success. This includes the construction of weighted and directed versions of the WWS time-varying network, among other refinements.

## Supplementary information

Supplementary Information
