# Supplementary material for: Predicting success in the worldwide start-up network
Source: Sci Rep. 2020 Jan 15;10:345. doi: 10.1038/s41598-019-57209-w (PMC6962148; doi:10.1038/s41598-019-57209-w)
Supplement: Supplementary file 1 — Supplementary Information [file 41598_2019_57209_MOESM1_ESM.pdf]

Supplementary Information for:
Predicting success in the worldwide start-up network

Moreno Bonaventura[1,2,†], Valerio Ciotti[1,2,†], Pietro Panzarasa[2]
Silvia Liverani[1,3], Lucas Lacasa[1], Vito Latora[1,3,4,5]
[1]School of Mathematical Sciences, Queen Mary University of London,
Mile End Road, E14NS, London (UK)
[2]School of Business and Management, Queen Mary University of London,
Mile End Road, E14NS, London (UK).
[3]The Alan Turing Institute, The British Library NW12DB, London (UK)
[4]Dipartimento di Fisica e Astronomia, Università di Catania and INFN, 95123 Catania (Italy)
[5]Complexity Science Hub Vienna (CSHV), Vienna (Austria)

S1 Data set: additional details **2**

S2 Construction of the *World Wide Start-up* (WWS) network **3**

S3 Analysis of the WWS network **4**

S4 Open-deals recommendation method **7**

S4.1 Success rate in open-deals lists 8

S4.2 Real investors performance is similar to random expectation 11

S4.3 Details on overall success rate 14

S5 Additional analysis **18**

S5.1 Closeness centrality in successful vs non-successful start-ups 18

S5.2 Different centrality measures are correlated 19

S5.3 Recommendation methods based on other centrality measures 22

S5.4 The effect of fading links 22
S5.5 Possible confounding factor 1: the effect of venture capital funds 26
S5.6 Possible confounding factor 2: number of employees 30
S5.7 Possible confounding factor 3: start-up geographical location 30

S6 From recommendation to prediction of start-up success: supervised learning approaches 32
S6.1 Logistic regression: the unbalanced case. 35
S6.2 Logistic regression: the balanced case 37

S1 Data set: additional details

Data were collected from the `crunchbase.com` Web API and were updated until December 2015. The data provided by the Crunchbase website are manually recorded and managed by several contributors (e.g., incubators, venture funds, individuals) affiliated with the Crunchbase platform. Moreover, the data are further enriched by Web crawlers that scrape the Web, on a daily basis, in search for news about IPOs, acquisitions, and funding rounds. To date Crunchbase is widely regarded as the world's most comprehensive open data set about start-up companies. It contains detailed information on organizations from all over the world and belonging to four categories, namely companies, investors, schools, and groups. Among schools there are 383 universities, including top-tier institutions such as Stanford University, the Massachusetts Institute of Technology (MIT), and many others. In addition to people's business activity, the data track information about their educational paths, and consequently their access to academic knowledge.

The total number of organizations listed at the date of data collection amounted to $530,604$. However, a large number of entries contained very limited information, no profile pictures, and

no employees' records. Accordingly, we needed to clean the data keeping only the organizations for which enough information was provided, and for which such information was reliable (see Section S2 for more details). This finally limited the number of organizations to $41,830$. For this work, all these organizations were included in the construction of the network. Notice however, that only organizations belonging to the category "companies" and, at the same time, younger than two years, have been included in the recommendation list. For each organization we extracted all the people included in the team (e.g., founders, advisors, board member, employees, alumni) and additional information such as details on firms' foundation dates, locations of the firms' headquarters, founding rounds, acquisitions, and IPOs. Organizations and people are uniquely identified by alphanumeric IDs. All data are time-stamped, and an accurate reconstruction of historical events was made possible by the use of trust codes, i.e., numerical codes provided by Crunchbase to indicate the reliability of a certain timestamp. The timestamps indicate the dates of foundation, funding rounds, acquisitions, and IPOs, as well as the start and the end times of job roles.

S2 Construction of the *World Wide Start-up* (WWS) network

We constructed a bipartite time-varying graph with $N_1 = 41,830$ nodes representing organizations distributed across 117 countries around the globe, $N_2 = 36,278$ nodes representing people, and $K_{12} = 284,460$ links between people and organizations. The graph is time-varying because each node and each link have an associated timestamp, representing, respectively, the time an organization was founded and the time a person was affiliated (and held a variety of roles) with a given organization. Notice that in the construction of the time-varying graph we retained only the timestamps whose trust code guarantees the reliability of the year and month. Additionally, we cleaned the data by solving and removing inconsistencies such as an employee's role starting at a date prior to the company's foundation. In these cases, we retained

the most reliable information according to the trust code value. Inconsistencies were removed by adopting a *strong self-penalising data cleaning strategy*. In particular, we did not make any assumption on dates, nor did we attempt to infer timestamps. As a result, we do not retain in the graph links whose timestamps cannot be determined in a reliable way. This approach to data cleaning strengthens the validity of our results because it ensures that companies do not gain higher positions in the closeness centrality rank score as a result of connections that were forged at subsequent dates to those incorrectly or only partially reported in the data set. In this way we avoid biases that could artificially inflate the success rate of the method, and accordingly our results can safely be seen as conservative lower bounds.

We then projected the bipartite time-varying graph onto a one-mode graph in which two companies are connected when they share at least one individual that plays or has played a professional role in both companies. Such a graph comprises $N_1 = 41,830$ companies and $K = 135,099$ links among them, and is here referred to as the *World Wide Start-up* (*WWS*) network. The projected graph is time-varying like the original bipartite graph: a link between any two companies is forged as soon as one individual with a professional role in one company takes on a role in the other company. Since the creation of these links denote intel transfer between companies, we realistically assume that such intel flow generates considerable know-how for the nodes receiving new links. Once created, the links are then maintained, since the know-how of a given company is not destroyed or removed.

S3 Analysis of the WWS network

For completeness, we have calculated a variety of quantities for measuring the characteristics of the structure of the WWS network. In particular, from 1990 to 2015, for every month, we have computed the number of companies (nodes) and links, and examined the partition of the WWS network into distinct connected components. A connected component of a network is a

subgraph in which any two nodes are connected to each other by at least one path [*15, 16*]. If the network has more than one component, one can identify the largest connected component (LCC), namely the component with the largest number of nodes. The countries highlighted in blue in Fig 1A (main text) are those that have at least one start-up that is part of the LCC of the WWS network. Fig 1C (main text) shows a rapid growth in the fraction of start-ups in the LCC, thus highlighting the tendency that companies have to establish new connections with one another and move toward the core of the network. Like many other real-world complex networks, the WWS network is characterised by a rich topological structure, a small average shortest path length ($\ell = 4.74$), and a high value of the average clustering coefficient, $C = 0.6$, as expected from the one-mode projection of a bipartite network [*15, 16*]. The value of the average shortest path length is similar to the one obtained for an equivalent Erdös-Renyi random graphs (*32*) with the same number of nodes and edges (ℓ^{random} = 4.17). However, the statistical features of the WWS differ from those characterising random graphs: the degree distribution approaches a power-law with an exponent greater than 2 (see Fig. S1, panel B), the assortativity coefficient (*31*) is positive, namely $\gamma = 0.11$ (see Fig. S1, panel A) and this result holds even if all venture capital firms are removed from the network (panel C). The clustering coefficient is significantly larger than the one obtained for a corresponding random network, $C^{\mathrm{rand}} = 0.00013$.

To offer a glimpse of the structure of the WWS network, in Fig. S2 we show the subgraph obtained by using the k-core decomposition technique and including only the nodes that belongs to the 10th shell. The k-core decomposition of a graph (*33–35*) is a technique that iteratively deletes nodes starting from the most peripheral ones (i.e., nodes with degree equal to 1) and progressively unveil the most central and interconnected core of the network. Nodes are assigned to a *core value* equal to k accordingly to the k-core subgraph to which they belong.

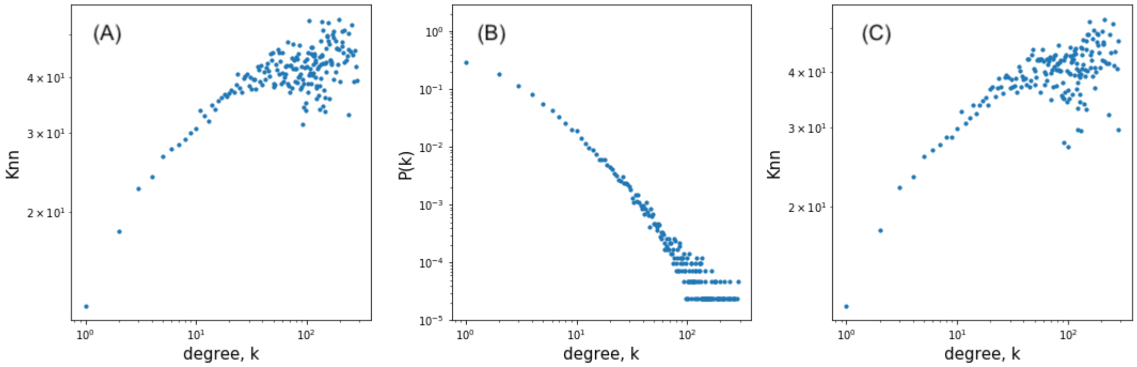

Figure S1: **Assortativity and degree distribution of the WWS network.** (A) Average degree of nearest neighbours k_{nn} for classes of nodes with degree k in the WWS. (B) The power-law degree distribution of the WWS network. (C) Similar to panel (A), but where all venture capital firms (see Section S5.5 for a list) have been removed.

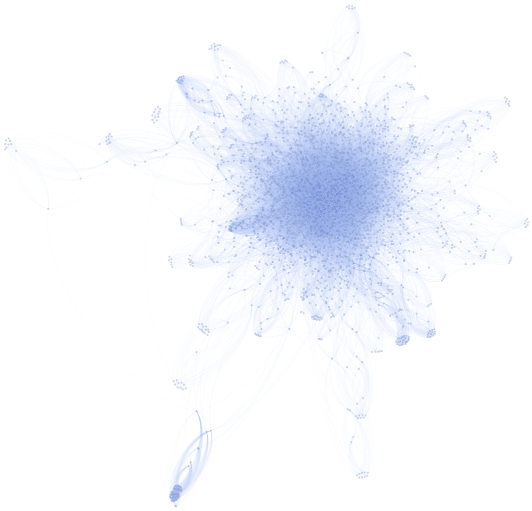

Figure S2: **Visualisation of the WWS network**. Owing to visualisation constraints, only the 10th shell of the k-core decomposition is displayed in the image. The graph shown here includes 8% of the nodes and 31% of the links in the complete WWS network.

S4 Open-deals recommendation method

Our working hypothesis is that companies with a central position in the network have higher exposure to knowledge and easier access to resources than companies with peripheral positions. If this is the case, centrally positioned companies will be better equipped to compete and have higher chances to survive, grow and flourish than peripheral ones. We have therefore used network centrality measures [*15, 16*] that capture the structural centrality of a node in a graph, with a view to identifying companies with a large long-term economic potential.

The concept of centrality and the related measures were first introduced in the context of social network analysis (*35*). the centrality of a company we have computed, on a monthly basis, its closeness centrality in the WWS network. Several other centrality measures have also been considered, and the results are reported in Section S5. The closeness centrality quantifies the importance of a node in the graph by measuring its mean distance from all other nodes. The closeness centrality $C_i(t)$ of a node i, $i = 1, 2, \ldots, N(t)$ is defined as:

$$C_i(t) = \frac{N(t) - 1}{\sum_j d_{ij}(t)}, \tag{S1}$$

where $N(t)$ is the number of nodes in the graph at time t, while $d_{ij}(t)$ is the graph distance between the two nodes i and j, measured as the number of links in the shortest path between the two companies. To account for disconnected components we used the generalisation of closeness centrality proposed in (*36*).

Our claim is that young start-ups with proportionally higher values of closeness centrality will have a higher likelihood to become successful in later years. This can be readily translated into several possible heuristics to provide recommendation for investing into a given start-up. Among other possibilities, we have considered the following recommendation method. For each month t, we ranked all the $N(t)$ companies according to their values of closeness centrality $C_i(t)$, such that the top nodes are those with the highest closeness. From the ranked lists we

then removed the companies that can reasonably be regarded as irrelevant deals to investors, i.e., those companies that had already been acquired, had already been listed in a stock market, or had received funding from other investors. The $N(t)$ companies retained in the analysis belong to the so-called *open-deals ranked list* at month t. Notice that, by definition, the open-deal list considers newly-established start-ups. As a matter of fact, incubators such as *500 Startups*, *Y Combinator*, *Techstars* or *Wayra* indeed target early-stage companies, i.e. they make risky investments on ideas and small teams without much of previous history. Their investment targets are therefore similar to the ones captured by our the definition of ‘open-deal list’, and it is easy to realize that predicting future positive outcomes of firms in such a set is more challenging than predicting future positice outcomes of more established firms.

Fig S3 shows an example of the procedure adopted. The companies highlighted in grey are those which, prior to December 2008, had not yet received funding, had not yet been acquired, or had no yet been listed in any stock market. These companies thus could be seen as investment opportunities at month t. Since we want to focus on early-stage companies, we also removed any company that was more than two years old.

S4.1 Success rate in open-deals lists

Each open-deals list in month t contains $M(t)$ successful companies ($0 \leq M(t) \leq N(t)$), i.e., those companies that have obtained, within a time window $\Delta t = 6, 7$ or 8 years since month t, a positive outcome. A positive outcome is here defined in terms of the occurrence of at least one of the following events: (i) the company makes an acquisition; (ii) the company is acquired; or (iii) the company undergoes an IPO. To each company in the open-deals list we then assigned the value of a binary variable, namely 1 if the company has achieved a positive outcome within the chosen time windows Δt, or 0 otherwise. Fig S4 shows an example of the monthly open-deals lists, in which names are replaced by their associated binary values. Notice

2008, December

Rank	All companies list	Open-deals list
1	Google	Airbnb
2	Apple	Uber
3	Facebook	Eventbrite
4	Intuit	...
5	Airbnb	
6	Digg	
7	Twitter	
8	Uber	
9	Eventbrite	
...	...	

Figure S3: **Example of the construction of the open-deals ranked list.** For each month of the observation period, all companies in the network were ranked according to their values of closeness centrality. Top-ranked nodes are those with the highest closeness centrality in the WWS network in the corresponding month. Only those companies (highlighted in grey) that had not yet received funding, had not yet been acquired, and had not yet been listed in the stock exchange market, were retained in the open-deals ranked list.

that the higher the number of ones in the top regions of the rankings, the better the performance of the recommendation method in predicting positive outcomes.

We focus on companies in the top positions of our open-deals recommendation list, and we indicate by $m(t)$ the number of companies in the Top 20 in month t that have obtained a positive outcome, i.e., the number of ones in the first $n = 20$ entries of the list. Notice that the same procedure has been repeated for the Top 50 companies ($n = 50$) to check for the robustness of results. The accuracy of the recommendation method is assessed by computing the success rate $S(t)$ defined as the ratio $m(t)/n$. How does this compare to a null model where network properties are not taken into account? If the open-deals lists were randomly ordered, the expected number of successful companies $m^{\text{rand}}(t)$ in e.g. the Top 20 ($n = 20$) would be given by the expected value of the hypergeometric distribution $\text{H}(N(t), M(t), n)$. In particular, the expected value of $m^{\text{rand}}(t)$ is $nM(t)/N(t)$ and thus the expected success rate is

Rank	Jan - '08	Feb - '08	Mar - '08	Apr - '08	May - '08	...
1	1	1	0	0	1	
2	1	0	1	1	0	
3	0	1	1	1	0	
4	0	1	1	1	1	
5	1	1	1	0	1	
6	0	0	1	1	0	
7	1	0	1	1	1	
8	1	0	1	1	0	
9	0	1	0	0	0	
10	0	1	0	0	0	
...	...	...	...	...	...	
$S^{Top\ 10}(t)$	5/10	6/10	7/10	6/10	4/10	

Figure S4: **Illustrative example of a monthly open-deals ranking.** Companies' names are replaced by the values of their associated binary variable, with a value equal to 1 indicating the achievement of a positive outcome. The last line reports the success rate $S^{\text{Top10}}(t)$ of companies in the Top 10 of our recommendation list.

$S^{\text{rand}}(t) = M(t)/N(t)$. Similarly, it follows that var$(S^{\text{rand}}(t)) =$ var$(m^{\text{rand}}(t))/n^2$.

Fig 2 (main text) and Fig S5 show that $S(t)$ (blue curve) is systematically much higher than $S^{\text{rand}}(t)$ (black curve), except during two short periods corresponding, respectively, to the dot-com bubble (1999-2001) and to the 2008 financial crisis. In both cases, the difference between $S(t)$ and $S^{\text{rand}}(t)$ becomes narrower, yet $S(t)$ always remains higher than $S^{rand}(t)$. Moreover, Fig S5 shows that these findings are robust against variations in the length of the time window (i.e., $\Delta t = 6, 7, 8$) and in the number of companies considered in the recommendation (i.e., Top 20 and Top 50).

The statistical significance of the results is assessed by computing the hypergeometric p-values, which give the probability of obtaining, by chance, a success rate equal to or greater than the one obtained with real data. Denoting as $P(\cdot)$ the probability mass function of $m^{\text{rand}}(t)$

we can compute the p-value at time t as:

$$p(t) = \sum_{k=m(t)}^{n} P(m^{\text{rand}}(t) = k).$$

The top charts in Fig 2 (main text) and in each panel of Fig S5 report the evolution of the p-values over time. Low p-values (< 0.05) are observed in most parts of the observation period. This suggests that the discrepancy between the success rate of the 20 top-ranked companies selected according to our recommendation method and the success rate of the same number of companies selected at random from the open-deals list is statistically significant. Conversely, high p-values are observed in correspondence of the downturns, thus indicating that in such periods the success rates predicted by our recommendation method could have been obtained also by chance.

S4.2 Real investors performance is similar to random expectation

It is important to highlight that, although the random expectation null model has mainly been introduced to assess whether our results are statistically significant, the performance of real investements is remarkably similar to the expected success rate in the null model. To illustrate this, a summary statistics of the Top 15 investors, according to the number of investments, is reported in Table S4.2 (data extracted from `crunchbase.com`). Notice that there is a great variability in investors performance, which reflects the variability in the type of investments. Highlighted in pink are those investors whose target complies with our definition of open-deal list. Incubators such as *500 Startups*, *Y Combinator*, *Techstars* or *Wayra* focus indeed their interest on very early-stage companies, i.e. they invest on ideas and small teams of entrepreneurs without much history. They make the most risky bets in the landscape of start-ups investments and their performance lies around 15%. Their investment target is very close to the type of companies that we have isolated in our definition of “open deals”. On the other end, large

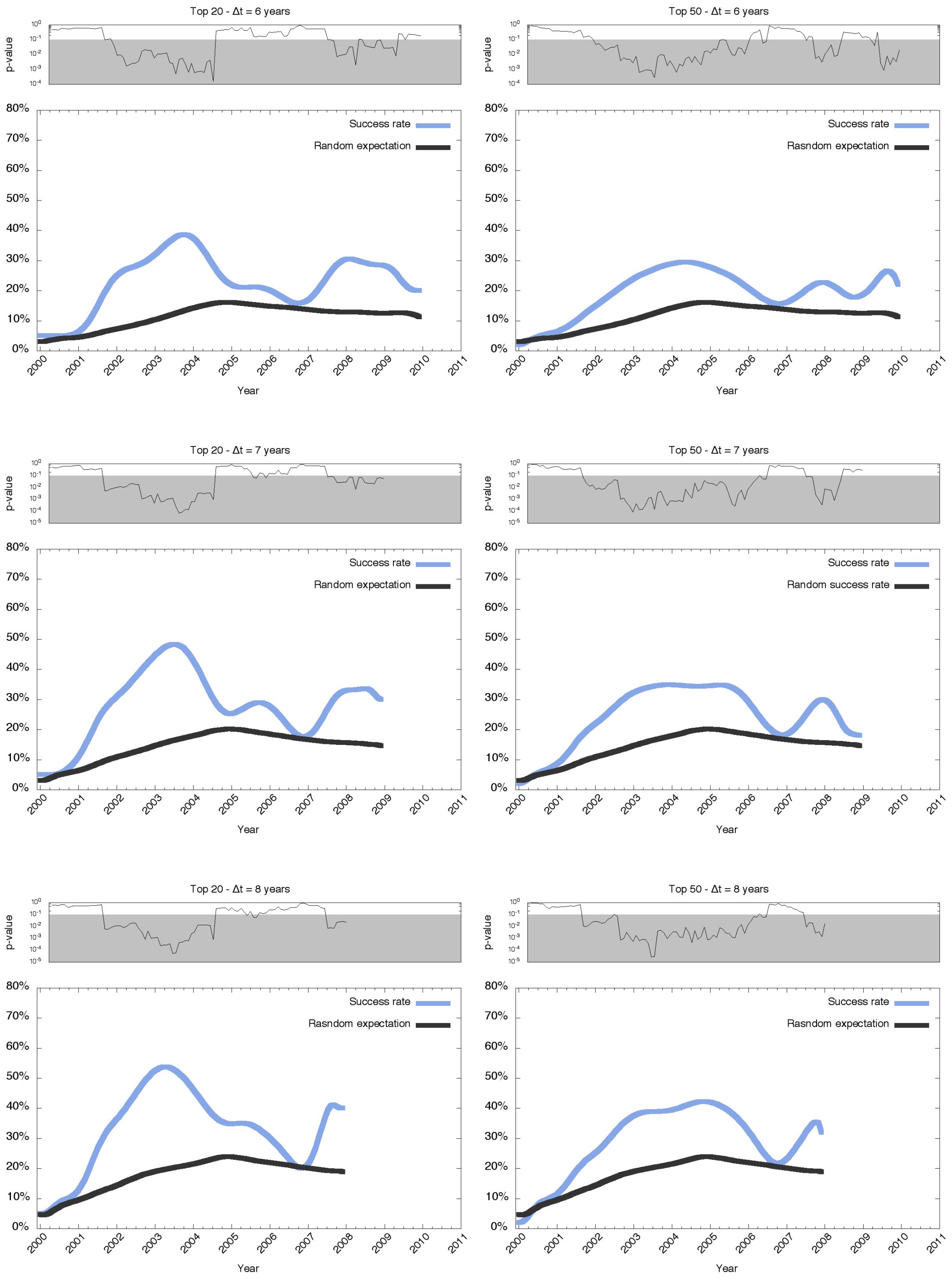

Figure S5: **The success rate of our recommendation method.** The success rate $S(t)$ of our method (blue curve) is compared to the expected success rate $S^{\mathrm{rand}}(t)$ associated with the recommendation of randomly selected companies (black curve). Different lengths, namely $n = 20, 50$, of the recommendation lists, and different time windows, i.e., $\Delta t = 6, 7, 8$ years, to assess the performance of a company have been considered. The statistical significance of the discrepancy between $S(t)$ and $S^{\mathrm{rand}}(t)$ is quantified through the associated p-values, shown in the top charts of each panel.

venture firms such as *Intel Capital*, *Accel Partners*, or *Goldman Sachs* invest in companies at later stage of maturity. They are interested in organizations with larger teams, that have already previously received funding, and they typically inject funds to boost a business that has already found a market fit and has history of revenues, customers, and other indicators of growth. The presence of quantitative indicators of growth allows large venture firms to perform a more objective evaluation of the company and its success potential, which in turn is reflected on higher investment performances.

**Investor**	**# investments**	**# successful investments**	**Success rate**
500 Startups	1022	153	**15%**
Y Combinator	953	154	**16%**
Intel Capital	744	313	40%
Start-up Chile	710	10	1.4%
Sequoia Capital	700	267	38%
New Enterprise Associates (NEA)	672	272	40%
SV Angel	600	258	43%
Techstars	549	95	**17%**
Brand Capital	537	80	14%
Accel Partners (Accel)	536	270	50%
Sos Ventures (SOSV)	493	17	3%
Wayra	476	11	**2%**
Kleiner Perkins Caufield & Byers (KPCB)	457	203	43%
Right Side Capital Management (RSCM)	449	44	10%
Goldman Sachs	410	209	50%

Table S1: Top 15 investment companies according to the number of investments made, along with the percentage of successful investments. Highlighted in pink are investors focused on very early-stage companies as those considered in our open-deal lists. The success rates of such investors are comparable to the random expectation null model, and much below the success rate obtained using our recommendation method.

In summary, while investors decide on which start-ups to invest through costly and labour-intensive screening processes, results confirm that the percentage of real investments that were deemed ‘successful’ is consistently similar to the success rate given by our random expectation model. In other words, state-of-the-art success rate is not much better than a random expectation null model. This means that any improvement upon the null model provides valuable informa-

tion. We conclude that our recommendation method based on centrality –whose success rate consistently exceeds random expectation over several periods– is a considerable improvement with respect to the state of the art.

S4.3 Details on overall success rate

To obtain an overall measure of the performance of our method, the success rate can be aggregated across the entire observation period. This can be carried out in two complementary ways leading to two different measures of the overall success, namely $\widetilde{S}_{\mathrm{I}}$ and $\widetilde{S}_{\mathrm{II}}$. Here we discuss and provide some details with regards to both measures.

The first measure of overall success rate, $\widetilde{S}_{\mathrm{I}}$, which is used in the main text, takes into account the total number of positive entries in the top positions in all open-deals lists, regardless of the specific companies that occupy those positions. In this way $\widetilde{S}_{\mathrm{I}}$ provides a measure of the overall goodness of the ranking across months, but it does not provide information about the number of unique companies correctly or wrongly identified as successful. As an example of the computation of $\widetilde{S}_{\mathrm{I}}$, let us consider the period starting in January 2000 and ending in December 2007, and the Top 20 companies (bottom-left charts in Fig S5). Such a period includes $\delta = 96$ months. The overall success rate $\widetilde{S}_{\mathrm{I}}$ is defined as:

$$\widetilde{S}_{\mathrm{I}} = \frac{\widetilde{m}_{\mathrm{I}}}{\widetilde{n}_{\mathrm{I}}},$$

where $\widetilde{n}_{\mathrm{I}} = 20 * \delta$ is the total number of entries in the Top 20 list across the δ months, and $\widetilde{m}_{\mathrm{I}} = \sum_t m(t)$, where the sum runs over all months in the observation period. To construct a null model to which we can compare these measures, we then proceed to randomly shuffling the entries in each open-deal list independently for each month and apply the same procedure (i.e., the null model makes a random sampling of the list without replacement). Accordingly, at month t we count the number of successful companies within the Top 20 and label it $m^{\mathrm{rand}}(t)$.

The expected total number of successful companies within all the Top 20 lists in this null model is thus given by:

$$\widetilde{m}_{\mathrm{I}}^{\mathrm{rand}} = \sum_t m^{\mathrm{rand}}(t),$$

and the corresponding variance is given by the sum of the variances in each month

$$\text{var}(\widetilde{m}_{\mathrm{I}}^{\mathrm{rand}}) = \sum_t \text{var}(m^{\mathrm{rand}}(t)),$$

where $\text{var}(m^{\mathrm{rand}}(t))$ denotes the variance associated to the random null model, i.e. the variance of the hypergeometric distribution. The expected overall success rate in the case of random ordering is then given by

$$\widetilde{S}_{\mathrm{I}}^{\mathrm{rand}} = \frac{\widetilde{m}_{\mathrm{I}}^{\mathrm{rand}}}{\widetilde{n}_{\mathrm{I}}},$$

and its standard deviation σ_{I} is

$$\sigma_{\mathrm{I}} = \frac{\sqrt{\text{var}(\widetilde{m}_{\mathrm{I}}^{\mathrm{rand}})}}{\widetilde{n}_{\mathrm{I}}}.$$

Figure S6 reports the overall success rate empirically found $\widetilde{S}_{\mathrm{I}}$ (blue bars), the overall success rate $\widetilde{S}_{\mathrm{I}}^{\mathrm{rand}}$ expected by chance (black dots), and its standard deviation (black error bars) for various values of Δt, and for different numbers of recommended companies (i.e., Top 20, 50, and 100).

The second measure of the overall success rate, $\widetilde{S}_{\mathrm{II}}$, does not simply capture the overall performance of the ranking-based recommendation method, but compares the number of unique companies in the Top 20s correctly predicted as successful by our method, across the entire observed period, against the number of successful companies that would be expected under random selection. In particular, this second measure of overall success is based on: (i) the total number $\widetilde{N}_{\mathrm{II}}$ of unique companies available in any month; (ii) the total number $\widetilde{M}_{\mathrm{II}}$ of unique companies that have achieved a positive outcome at any time since their foundation up to 2015; (iii) the number $\widetilde{n}_{\mathrm{II}}$ of unique companies included in all Top 20 rankings in any month; and (iv)

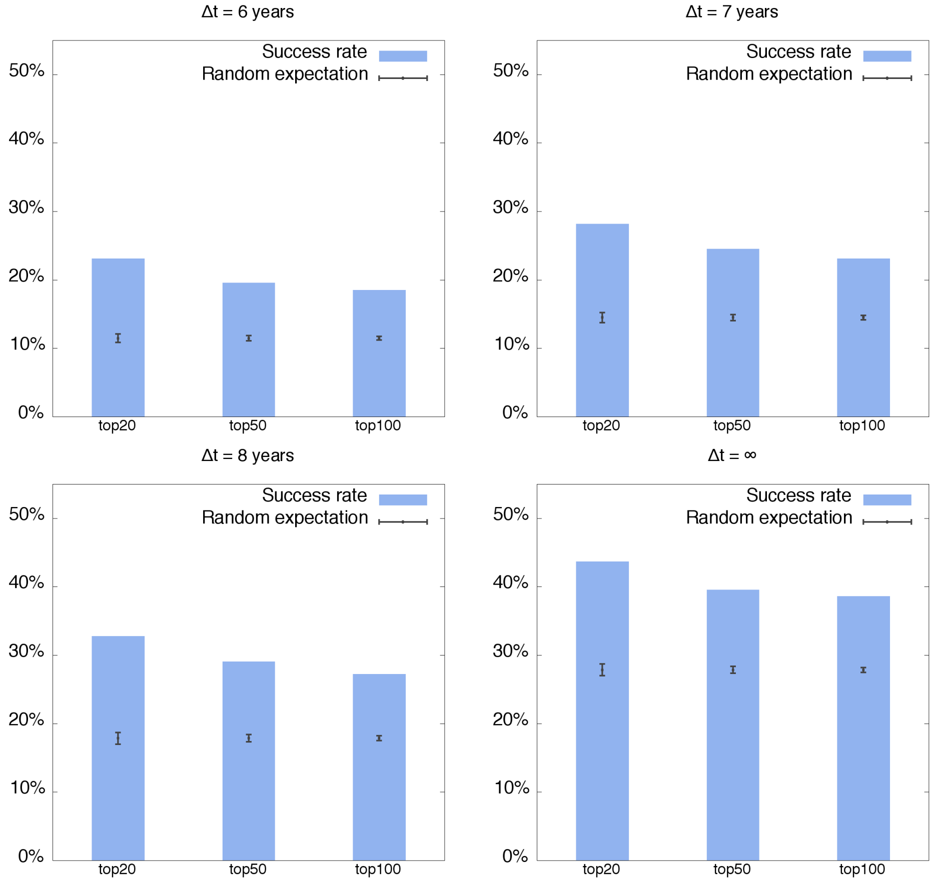

Figure S6: **Observed and randomly expected success rates**. The overall success rate empirically found $\widetilde{S}_{\mathrm{I}}$ (blue bars), the overall success rate $\widetilde{S}_{\mathrm{I}}^{\mathrm{rand}}$ expected by chance (black dots), and its standard deviation (black error bars), for various values of Δt and lengths of the list of top-ranked companies (i.e., Top 20, 50, and 100).

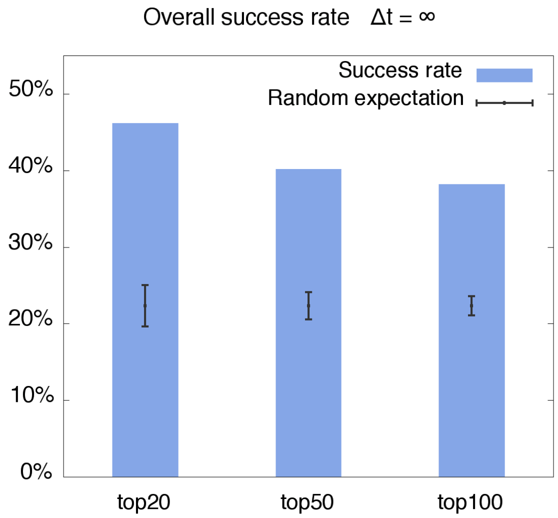

Figure S7: **Observed and randomly expected success rates.** The overall success rate $\widetilde{S}_{\mathrm{II}}$ (blue bars) obtained through the second method of aggregation assessed against the overall success rate $\widetilde{S}_{\mathrm{II}}^{\mathrm{rand}}$ expected by chance (black dots), and its standard deviation (black error bars), for various lengths of the list of top-ranked companies (i.e., Top 20, 50, and 100).

the number $\widetilde{m}_{\mathrm{II}}$ of unique companies, listed in all Top 20 rankings, that have achieved a positive outcome at any time since their foundation up to 2015.

Notice that, in this way, each company contributes only once to the evaluation of the success rate. Therefore, the probability of finding exactly $\widetilde{m}_{\mathrm{II}}$ successful companies in any ranking of Top 20 (50, or 100) is given by the hypergeometric function $\mathrm{H}(\widetilde{N}_{\mathrm{II}}, \widetilde{M}_{\mathrm{II}}, \widetilde{n}_{\mathrm{II}}, \widetilde{m}_{\mathrm{II}})$. The success rate shown in Fig S7 is computed as $\widetilde{S}_{\mathrm{II}} = \widetilde{m}_{\mathrm{II}}/\widetilde{n}_{\mathrm{II}}$, while the success rate $\widetilde{S}_{\mathrm{II}}^{\mathrm{rand}}$ in the case of the null model is given by $\widetilde{S}_{\mathrm{II}}^{\mathrm{rand}} = (\widetilde{M}_{\mathrm{II}}/\widetilde{N}_{\mathrm{II}})$. Fig S7 reports also the error bars of the success rate computed as the standard deviation of the hypergeometric distribution.

While the first index of overall performance assesses the average goodness of the ranking, the second index measures only the number of companies correctly identified as successful across the entire observation period. The two aggregation methods produce comparable results, and achieve a substantial success rate of about 40% in the case of $\Delta t = \infty$. Moreover, in

both cases, the success rate found in reality and the one expected by random chance are very different, and their discrepancy is always statistically significant with p-values smaller than 10^{-5}.

S5 Additional analysis

S5.1 Closeness centrality in successful vs non-successful start-ups

To have a better understanding of how closeness centrality is distributed among start-ups, in Fig S8 we compare the estimated frequency histograms of closeness centralities rescaled ranking. To obtain the rescaled ranking, in each calendar month we calculated the closeness centrality of each firm in the global network and ranked all firms in terms of their centrality, what gives an 'absolute rank' for each firm. We then extract those firms which belong to the open-deal list, and re-rank them accordingly (so that the firm with top ranking acquires a rank 0 in the open-deal ranking, the second acquires rank 1, and so on). The rescaled ranking is then defined as the ratio between the open-deal-rank and the maximum absolute-rank of open-deal companies at a given month. Thus, the firm with the highest position (i.e., zero ranking) maintained the same value (i.e., zero) in the rescaled ranking. By contrast, firms at lower positions were assigned rescaled values approaching 1 as their ranking approached the highest value (i.e., the lowest position). Such a rescaling thus enables to appropriately compare firms characterised by different values of centrality, obtained in different networks and at different calendar times. In order to smooth out the data, a binning has been performed in the x axis (bin size of 0.005). We notice that histograms are non-overlapping, and that there is a net overabundance of start-ups with a positive outcome (successful) closer to the top of the ranking. In other words, start-ups which are higher in the centrality rankings (i.e. small values of closeness centrality ranks) have statistically a higher chance of positive economic outcomes. This confirms that rankings based on closeness centrality are indeed informative of a start-up long-term success and can then be

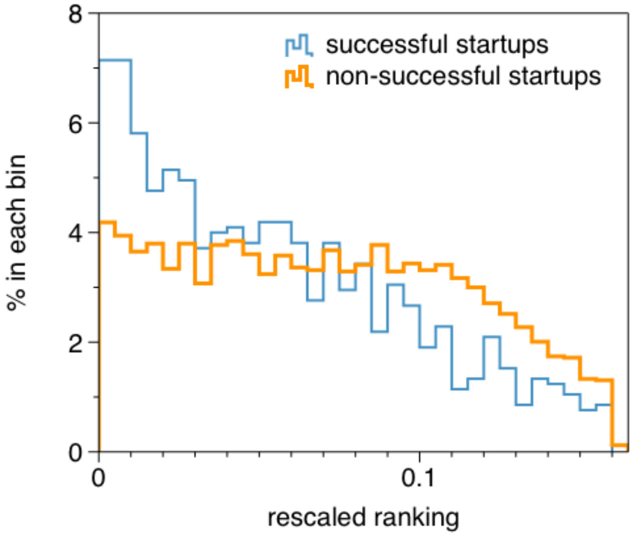

Figure S8: **Closeness centrality distributions.** Histograms of closeness centrality rescaled rankings (in bins of 0.005, see the text) for start-ups which will have a positive outcome (blue) and for which no positive outcome occurs (orange). Successful start-ups have statistically lower rescaled rankings (i.e. higher centralities) than non-successful ones. For every start-up, only the value of closeness centrality collected in the last month of observation has been used.

used to inform recommendation.

S5.2 Different centrality measures are correlated

We have considered closeness centrality as our primary measure of network centrality. Closeness centrality is based on the lengths of shortest paths in the network. However, the structural centrality of a node in a network can be quantified by different network metrics, either global such as closeness and betweenness, and local as the degree centrality (*35*). *Closeness centrality* of a node (Eq.S1) characterises the overall distance between that node and the rest of the nodes in the network, such that the lower that overall distance, the higher this measure, and hence the more central this node is.

On the other hand, the *betweenness centrality* $b_i(t)$ of a node i when the network is observed at

a given time t is given by

$$b_i(t) = \frac{1}{(N-1)(N-2)} \sum_{j=1, j\neq i}^{N} \sum_{k=1, k\neq i,j}^{N} \frac{n_{jk}(i;t)}{n_{jk}(t)}, \tag{S2}$$

where $n_{jk}(t)$ is the total number of shortest paths between nodes j and k whereas $n_{jk}(i)$ is the number of shortest paths between j and k that actually go through i. This measure was introduced by Freeman to quantify the fact that communication travels *just* along shortest paths, and so a node i is more 'central' the more shortest paths among pairs of nodes in the network go through it.

While both closeness and betweenness are measures of centrality based on shortest paths, one can also think of a node being central if it acquires many edges over time –i.e. acquiring intel from several other companies–. To account for this we may resort to use *(normalised) degree centrality* $d(i)$, defined as

$$d_i(t) = \frac{k_i(t)}{k_{max}(t)}, \tag{S3}$$

where $k_i(t)$ is the degree (number of links) of node i and $k_{max}(t)$ is the largest degree in the network at that particular time snapshot.

Consequently, the centrality of a start-up in the WWS network can be measured in many alternative ways. In this section we will show that the choice of using closeness centrality is not only supported by theoretical arguments based on employees' mobility and intel flows among companies, but it also a robust choice as other alternative measures produce similar results.

To validate robustness, for each start-up in the open-deal list across time we have computed additional centrality measures, namely degree and betweeness centrality (*30*), and computed to which extent all three possible measures of centrality are correlated. More concretely, we consider all start-ups in the open-deal list for which (i) we have data of the three centralities over at least 3 of the 24 months forming the observation window, and for which (ii) closeness

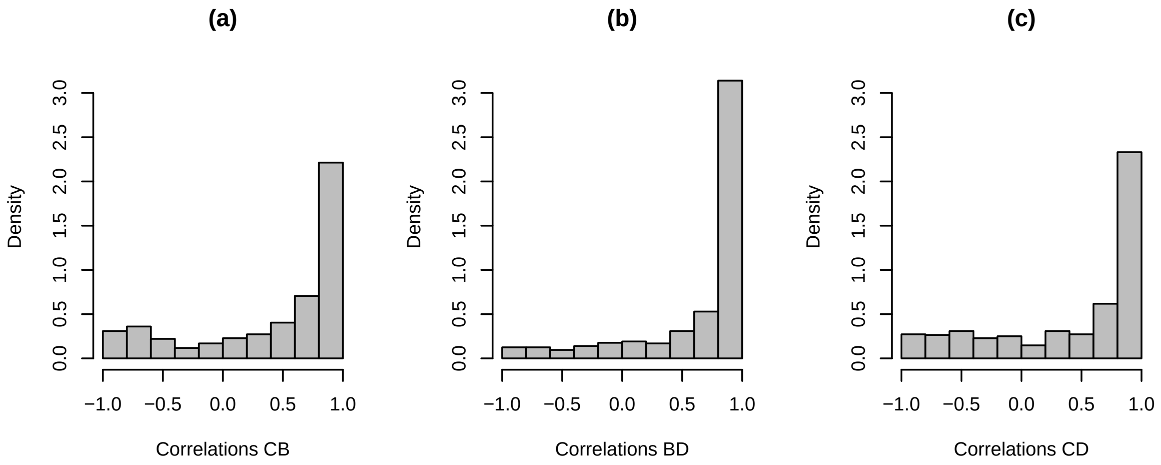

Figure S9: **Centrality measures are correlated.** Frequency histograms of the Pearson correlation coefficients between (a) rescaled closeness and rescaled betweeness centrality, (b) rescaled betweeness and rescaled degree centrality, and (c) rescaled closeness and rescaled degree centrality. We find that centrality measures are systematically correlated between each other, so the choice of using closeness centrality as the centrality measure under analysis is robust.

and betweeness centralities are defined. For each firm, we then compute the Pearson correlation coefficient between the monthly sequence of each pair of (rescaled) centrality measures. We do this for all firms and we then construct the frequency histogram of the Pearson correlation coefficients. Results are reported in Fig. S9. Interestingly, we find that the three measures are in general well (pairwise) correlated. We conclude that the choice of a particular type of global centrality measure, such as closenness, is a robust choice as other global structural indicators based on a different use of shortest paths and, to a minor extent, also local measures such as the degree are correlated with the closeness in the case of the WWS network under analysis in this work. Hence, focusing on closeness centrality is a robust choice. In the next subsection we round-off this validation by exploring results of our recommendation method using either betweenness or degree centrality as the key network indicator, and will show that success rates of the recommendation method are similar in all three cases.

S5.3 Recommendation methods based on other centrality measures

To further complement the correlation analysis of the previous subsection, here we focus on recommendation methods based on centrality measures other than closeness. Results are summarised in Fig.S10 for averages over the entire period, and in Figs.S11 and S12 for monthly analysis. In every case we find that the results are qualitatively similar whether we use closeness, betweeness or degree centrality, with success rates systematically larger than random expectations (and therefore larger than the actual perfomance of accelerators and investors focusing on early-stage start-ups).

S5.4 The effect of fading links

The mobility of workers from one company to another creates an intel flow between companies. Our working hypothesis is that companies receiving employees increase their fitness by capitalising on the know-how the employee is bringing with him/her. Such microscopic dynamics is thus captured and modelled by the creation of new edges at the level of the network of start-ups. As a consequence, companies which are perceived at the micro scale as appealing opportunities by mobile employees will likely boost their connectivity and therefore will acquire a more central position in the WWS network. An important underlying assumption is that, once a link is created, it will remain in the network indefinitely, so that the company that has received the intel keeps it and builds on this intel forever. Conversely, considering the possibility of removing links (or actually fading their strength) some time after their creation, would actually be equivalent to assume that companies can lose the know-how they have acquired, something which is less likely to occur. Accordingly, allowing links to fade or be removed with time in the construction of the time-varying WWS network should lead to recommendations on the positive economic outcome with much lower success rates than those obtained from a network where know-how is not artificially removed. To check for this case, we have first build the WWS (for

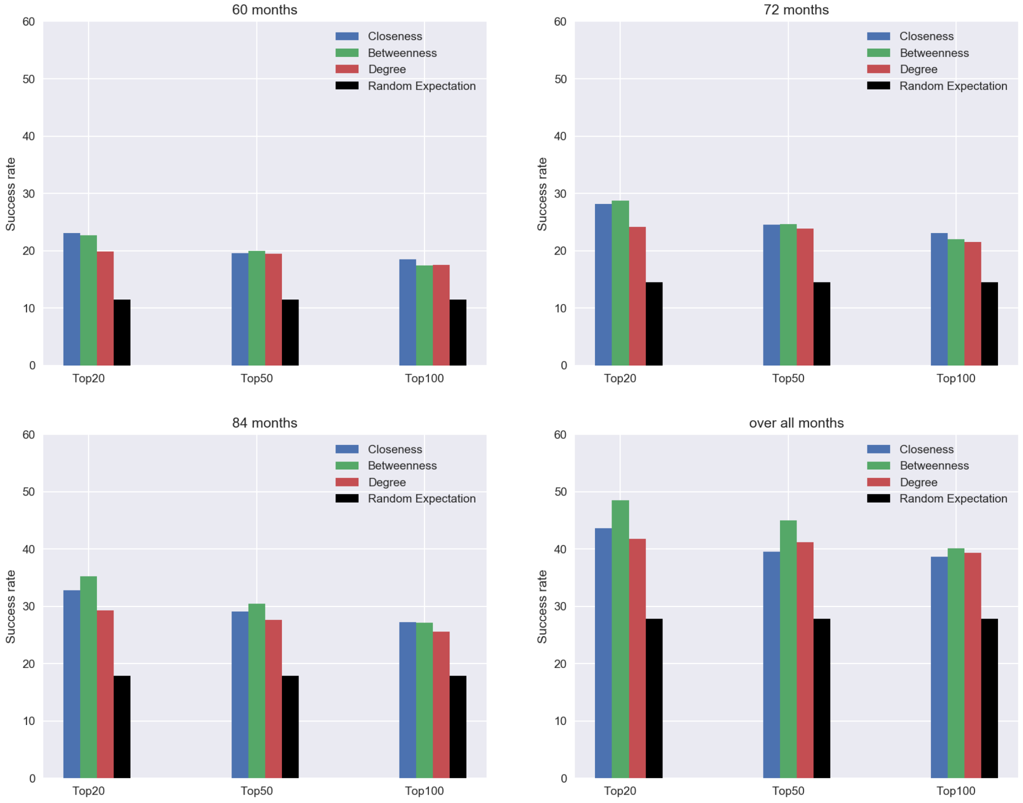

Figure S10: **Overall success rate of recommendation methods based on different centrality measures.** Comparison of recommendation methods focusing on the top 20, top 50 and Top 100 rankings, for $\Delta t = 5, 6, 7$ and ∞ years, using different centrality measures. Closeness, degree and betweeness centralities perform similarly, and recommendations based on either of these measures are systematically superior to a random expectation model, with overall success rates which are systematically larger than in the null model.

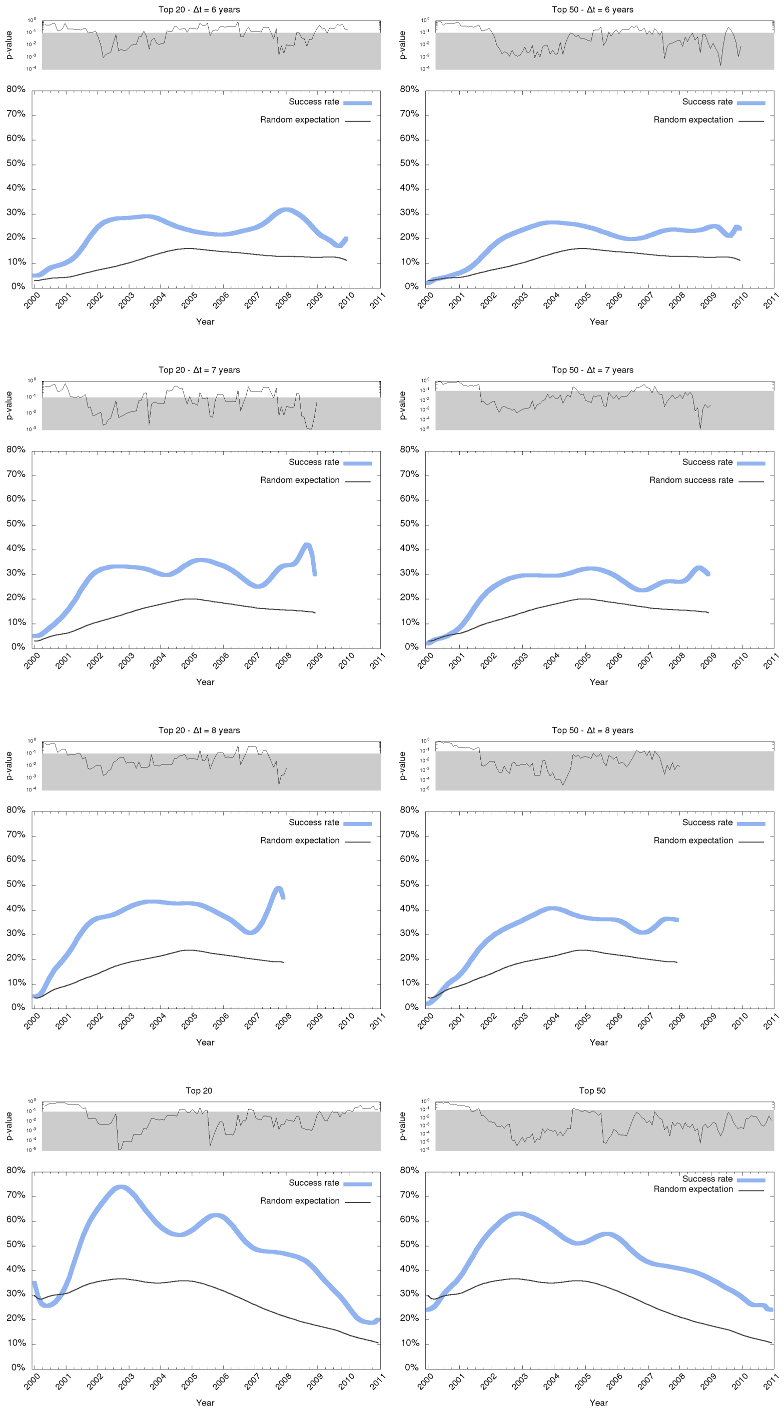

Figure S11: **Monthly success rate of recommendation methods based on betweeness centrality.** Recommendation methods focusing on the Top 20 and Top 50 ranked start-ups in the open-deal list, for $\Delta t = 5, 6, 7$ and ∞ years, using betweeness centrality instead of closeness centrality. Results are qualitatively similar and the monthly success rate of recommendations based on betweenness is systematically superior to that of a random expectation model.

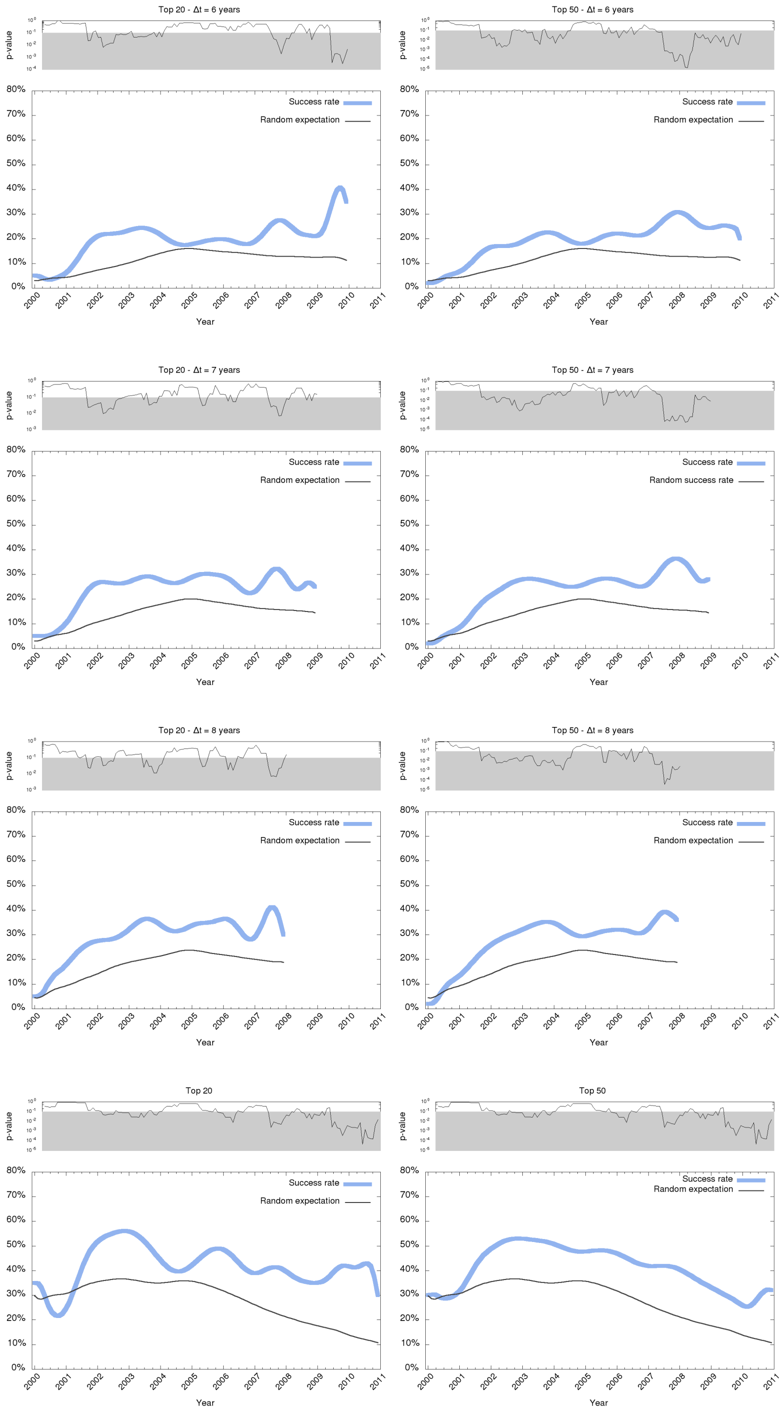

Figure S12: **Monthly success rate of recommendation methods based on degree centrality.** Recommendation methods focusing on the Top 20 and Top 50 ranked start-ups in the open-deal list, for $\Delta t = 5, 6, 7$ and ∞ years, using degree centrality instead of closeness centrality. Results are qualitatively similar and the monthly success rate of recommendations based on degree centrality is systematically superior to that of a random expectation model.

each month) from January 1990 to December 1999. Then, starting from January 2000 onwards, for each month all connections older than 10 years are removed from the network. Closeness is then evaluated each month as described in the recommendation method. A similar analysis is also performed for 5-year fading instead of 10-year fading, with very similar results.

In Fig.S13 we compare the overall success rate for the 5-year fading case (red bars) to our standard recommendation method based on a WWS network that does not allow link fading. Results show that a recommendation method with fading links systematically fails. In fact it works even worse than a random null model, in good logical agreement with our previous discussion. For completeness, a comparison of the two methods is also considered for the monthly success rates in Fig.S14. Results are consistent with those obtained for the overall success rate.

All these results strengthen our working hypothesis that the intel flow across start-ups is well captured by node centrality in the WWS network.

S5.5 Possible confounding factor 1: the effect of venture capital funds

A first possible confounding factor is the presence of venture capital funds, i.e. the fact that the presence of these nodes in the network might enhance the closeness centrality of start-ups. In order to assess the role played by venture capital funds in the effective centrality of different start-ups, we have performed an experiment where we remove all venture capital funds from the world start-up network, and subsequently have recomputed closeness centrality values for each start-up in the open-deal list. Concretely, we extracted from `CrunchBase.com` a list of 101 companies that are labelled as venture capital firms see Table S5 for details.

Accordingly, in this experiment we create the WWS network but not include those nodes in the network (and all the connections they bring with them). Closeness centrality is then evaluated

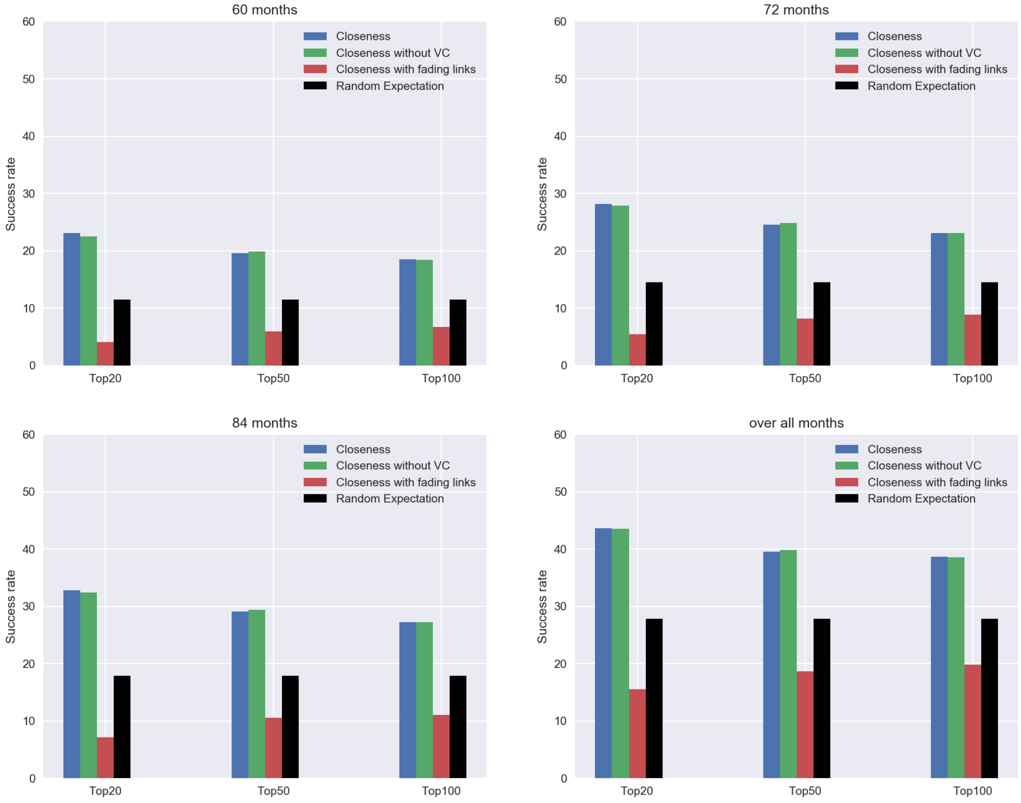

Figure S13: **Effects of fading links and removal of venture capital funds.** Success rate of recommendation methods focusing on the Top 20, Top 50 and Top 100 rankings, for $\Delta t = 5, 6, 7$ and ∞ years. The standard case of closeness centrality from the original network (blue bars) is compared to closeness centrality in a case where the links of the WWS are allowed to fade over time (red bars), and to closeness centrality in a situation where all venture capital funds (VC) have been removed from the WWS network (green bars). Results from the null model are plotted in black bars.

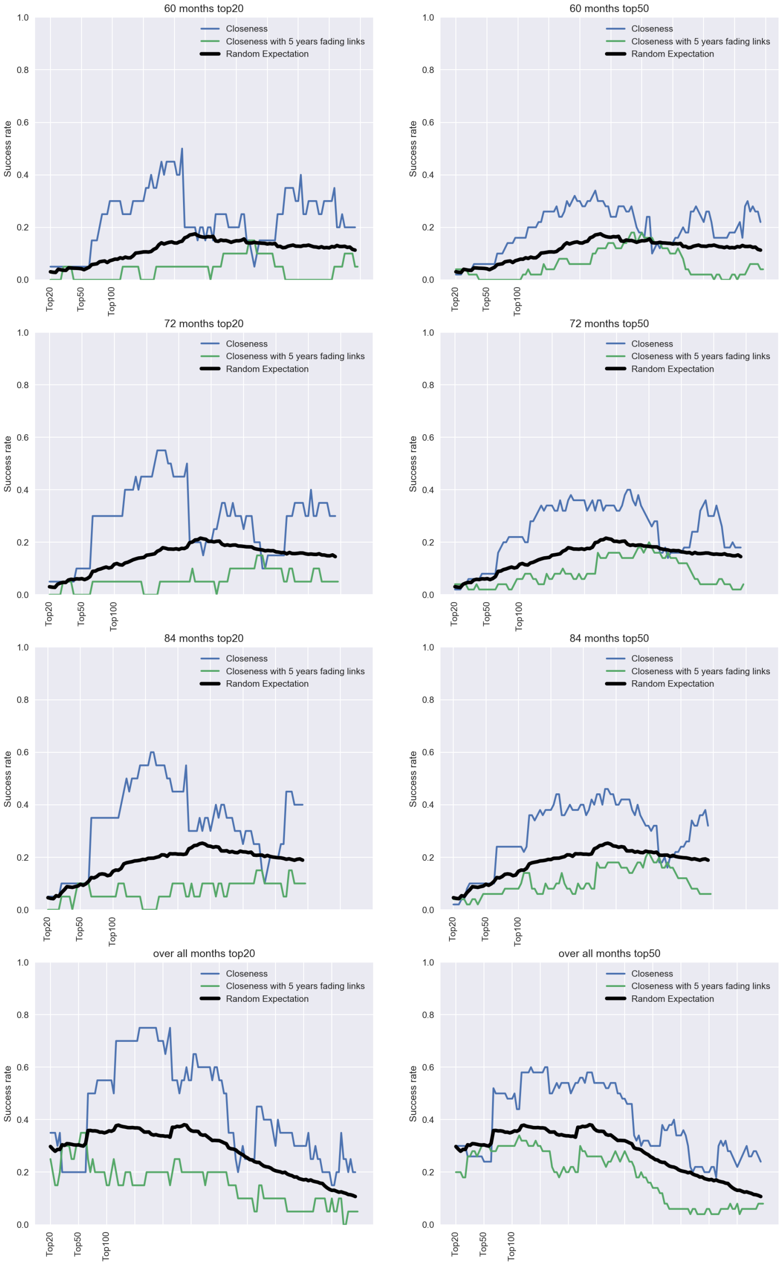

Figure S14: **Effect of fading links on monthly success rates.** Systematic comparison of the monthly success rate of a recommendation based on the closeness centrality from the original network and in a case where the links of the WWS are allowed to fade over time. In the latter case success rates drop below the random null model.

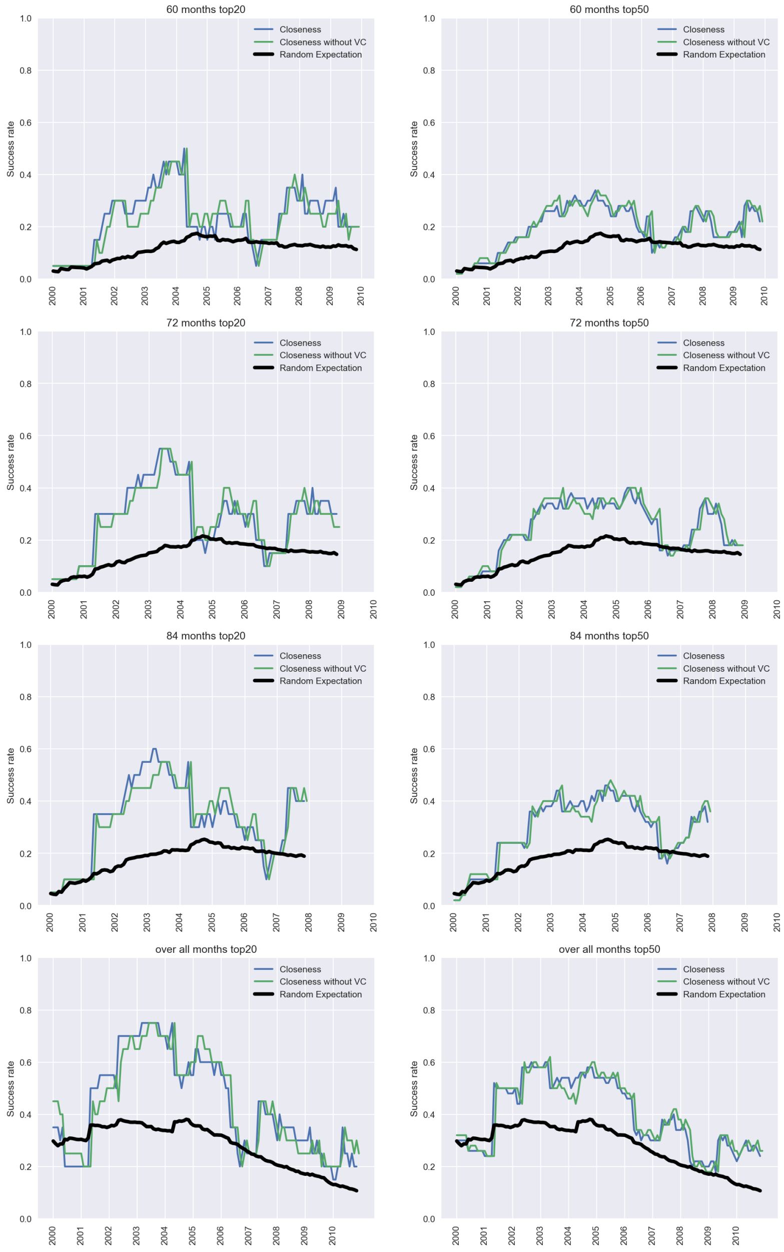

Figure S15: **Comparison of monthly success rate of recommendation method based on a worldwide start-up network with and without venture capital funds.** Monthly success rate of the recommendation method focusing in the Top 20 and Top 50, for $\Delta t = 5, 6, 7$ and ∞ years, comparing the original case to the case where all venture capital funds have been removed from the worldwide start-up network. Results are essentially identical to the ones obtained in the original case, hence confirming that the topological presence of venture capital funds is not a confounding factor.

each month as described in the recommendation method. Results of overall success rate are shown (green bars) in Fig.S13 while monthly success rates are compared in Fig.S15. The success rate of the recommendation method based on this quantity is consistently similar to the one found in the case where venture capital funds are not removed from the original network, hence confirming that the topological presence of venture capital funds is not a confounding factor.

S5.6 Possible confounding factor 2: number of employees

A second possible confounding factor or hidden predictor is the start-up size (e.g., number of employees). To assess this possibility, we have conducted a number of experiments. Initially, we explored start-up size (number of employees) instead of topological network centrality as the informative predictor, and built a recommendation method based on that metric. Results are shown in the left panel of Fig.S16, confirming that number of employees is not informative of the start-up success likelihood.

Additionally, we have also checked the recommendation method (based on closeness centrality) when only the subset of open-deal start-ups with a fixed number of employees is considered. Since the most frequent size is a start-up with a single employee, we extract the subset of all start-ups with only one employee. Monthly success rates of the recommendation method are shown in the right panel of Fig.S16. These results confirm that start-up size is not a counfounding factor and that number of employees is indeed not an informative variable that determines future success.

S5.7 Possible confounding factor 3: start-up geographical location

A third possible confounding factor is the geographical location of each of the start-ups. To account for this, we have replicated our analysis (originally performed at a worldwide scale)

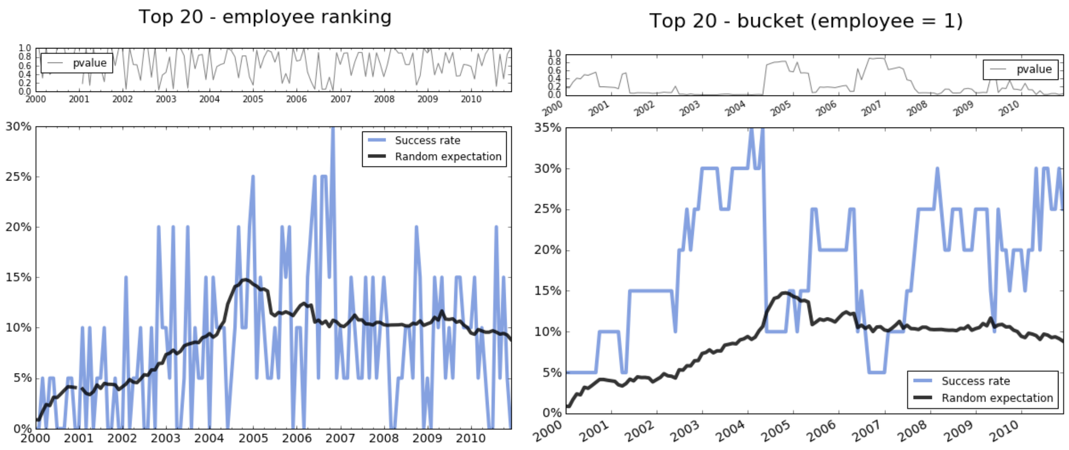

Figure S16: **Accounting for start-up size.** (Left panel) Monthly success rate of an hypothethical recommendation method *based only on start-up size* (number of employees), focusing in the Top 20 start-ups in the open-deal list. Results wildly fluctuate above and below a random expectation model, and p-values safely conclude that the number of employees is not informative of the start-up success likelihood. (Right panel) Monthly success rate of the recommendation method based on closeness centrality, focusing in the Top 20 start-ups from a subset of the open-deal list *gathering only start-ups with a single employee*. Results are qualitatively similar to the ones obtained without conditioning for start-up size, and suggest that start-up size is not a confounding factor.

in five geographically separated regions, by dividing open-deal start-ups in five subsets: California, United Kingdom, New York, Texas and Israel. Results for the monthly success rate of our recommendation method are plotted in Fig.S17. While results are more noisy than for the worldwide setup, we can confirm that for every case the recommendation method based on closeness centrality is above the random expectation.

S6 From recommendation to prediction of start-up success: supervised learning approaches

The recommendation method proposed in the main text is based on the working hypothesis that start-ups with higher closeness centrality rankings are more likely to experience a economic successful outcome in the future. We have provided theoretical foundation to our research hypothesis at the microscopic level, and then heuristically validated our recommendation lists on a monthly basis, obtaining results that are significantly better than those obtained by a random expectation model.

However, strictly speaking, a recommendation method is not a true prediction method, as we are not predicting the outcome of each and every start-up in the open-deal list (either to the successful or to the non-successful category). To bridge this gap, in this section we consider different types of prediction models which can indeed truly "predict" the positive outcome of a start-up, i.e. they can classify whether a given start-up will have a positive outcome or not.

All models are initially based on a sample including $5,305$ firms. These are the firms that have been in the open-deal list for at least one month, and can therefore be suggested as potential investment opportunities. Each firm is then observed over a period of 24 months, or until when it has experienced a "successful" event (positive economic outcome) if this event occurs before the end of the 24 months period. Notice however that in the greatest majority of cases firms were observed for 24 months. For this experiment note also that we are aggregating all the firms in

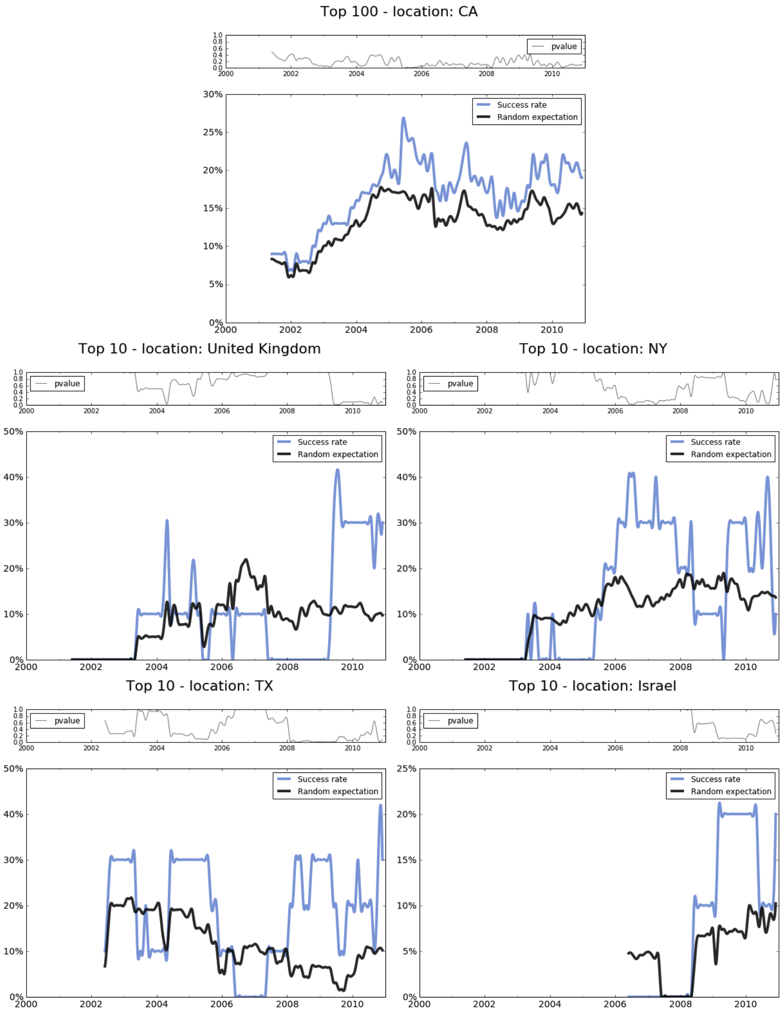

Figure S17: **Accounting for spatial location.** Monthly success rate of recommendation method on five geographically separated regions.: California, United Kingdom, New York (US), Texas (US) and Israel. For the California ecosystem we considered the Top100 ranking whereas for the other four (smaller) ecosystems we only considered the Top 10. Results are more noisy by qualitatively similar to the ones obtained in the original case, hence confirming that spatial location is not a confounding factor.

open-deal list in our database: not all of them are observed in the same time, e.g. one firm can be observed for the 24 months starting in January 2000, another firm can be observed for the 24 months starting in June 2004, etc. In other words, month $t = 1$ for a given firm does not necessarily matches the actual date of month $t = 1$ for another firm, we are simply recording the temporal evolution of different start-ups which appear in open-deal lists at different times in the period ranging from 1990 to 2008.

Then, for each of the $5,305$ firms, we conclude that a firm has experienced a "successful" event (a positive economic outcome) at month t if, within a time window of $\Delta t = 6, 7$ or 8 years since month t, one of the following events takes place: (i) the firm makes an acquisition; (ii) the firm is acquired; or (iii) the firm undergoes an IPO. Accordingly, each firm receives a unique class label (either successful with class label '1', if at any time $t + \Delta t$ the start-up experiences a successful event, or not-successful with class label '0' otherwise).

Overall, this data set enables supervised learning (classification), as it consists of a large number of samples (the firms in the open-deal list), each of them described by a set of features (a vector of centrality measures over the whole observation window), and each of them being labelled by a class label.

We will use logistic regression as our supervised learning model. A logistic regression model links the probability of success of a start-up to a linear combination of predictors. More precisely, a logistic regression model is traditionally given by:

$$\log\left(\frac{p_i}{1-p_i}\right) = \boldsymbol{\beta}^{\mathrm{T}}\mathbf{X}_i + \varepsilon_i \tag{S4}$$

where p_i is the probability of success of the i-th start-up, $\mathbf{X}_i$ is the vector of predictors, $\boldsymbol{\beta}^{\mathrm{T}}$ is the (transposed) vector of parameters, which are estimated when the logistic regression model is fitted, and ε_i are the errors, which are assumed to be independent, identically-distributed

Normal random variables. Rearranging terms, we have

$$p_i = \sigma(\boldsymbol{\beta}^{\mathrm{T}}\mathbf{X}_i + \varepsilon_i),$$

where $\boldsymbol{\beta}^{\mathrm{T}}\mathbf{X}_i + \varepsilon_i$ is a linear combination of predictors with additive noise term and $\sigma(x) = 1/[1 + \exp(-x)]$ is the so-called logistic function. In essence, the term $\boldsymbol{\beta}^{\mathrm{T}}\mathbf{X}_i + \varepsilon_i$ is akin to a linear regression on the predictors, and the logistic function is used to force the outcome to be equal to 0 or 1: if $p_i < c$, the class 0 is assigned, and for $p_i > c$ the class 1 is assigned, where the threshold c is indeed another parameter that can be trained by the algorithm. Once the parameters are estimated, logistic regression can be used to predict the probability of success of new start-ups.

In what follows we consider two scenarios. In the first case, we define prediction models that do not consider the time evolution of centrality measures for each firm and only use instantaneous values of the firm's closeness centrality: these models will be closer in spirit to the recommendation method. In a second case, we enrich the predictor set by adding predictors summarising the time evolution of the firm's centrality over the observation period (to assess whether this factor is informative) as well as similar quantities extracted from different centrality measures.

S6.1 Logistic regression: the unbalanced case.

Here we use the ROC (receiver operating characteristic) curve to assess the efficacy of this binary classification algorithm to choose the optimal threshold based on our tolerance for false negatives and desire for true positives. We initially have used only the last value of the (rescaled) closeness centrality of a start-up over the observed period as the single predictor, in order to try to match the conditions of our recommendation method where only instantaneous information is used. The estimation and prediction steps above have been repeated 1,000 times, leaving out 10% of the data set (Monte Carlo cross-validation). Averaging over the prediction results, we

obtain the confusion matrix reported in Table S2 (left panel), together with the confusion matrix expected for a random classifier operating on the same data set (right panel).

ACTUAL		predicted		RANDOM		predicted	
		failure	success			failure	success
**true**	failure	0.43	0.34	**true**	failure	0.593	0.177
	success	0.11	0.13		success	0.177	0.053

Table S2: (Left) Confusion matrix for a logistic regression based on the last value of closeness and on the mean closeness over the entire period in the unbalanced case. Averages over 1,000 repetitions of the Monte Carlo cross-validation leaving out 10% of the data. (Right) Corresponding confusion matrix expected for a random classifier in the same unbalanced case.

Classical ways to assess the prediction performance include the evaluation of *accuracy*, defined as the total percentage of correctly identified samples, *sensitivity*, defined as the percentage of successful start-ups correctly predicted by the classifier over the percentage of true successful start-ups, and *precision*, defined as the percentage of successful start-ups correctly predicted by the classifier over the total percentage of start-ups which are predicted as successful by the classifier. The *F1 score* is the harmonic average of precision and sensitivity. Depending on the context, it might be desirable for a classifier to have high sensitivity or precision, and when both quantities are relevant then the F1 score is typically used to assess model selection. In our case the sensitivity is the relevant quantity to look at if we want to maximise the detection of successful start-ups, whereas the precision is important if we want to make sure that all the start-ups classified as successful will be successful. In other words, the first performance indicator can be the one of relevance for an investment company with unlimited budget, while the precision can be of interest to an investment company with limited budget.

The values obtained for the different indicators are reported in Table S3. The *F1 score* –which trades off sensitivity and precision– shows that the predictions on whether any start-up in the open-deal list will have a positive outcome are systematically better than those of a benchmark given by a random classifier. Note that the problems with the accuracy are due to

the fact that our two classes are unbalanced, and this can affect the usefulness of this indicator. We will come back to this point in the next subsection.

We have also experimented by including additional features of the evolution over time of the closeness centrality as predictors in the logistic regression model. Interestingly, our results did not improve significantly, suggesting that it is not necessary to use temporal evolution of centrality measures, and thus confirming the validity of the recommendation method. This observation will be further explored in the next subsection.

	**Accuracy**	**Sensitivity**	**Precision**	**F1 score**
Unbalanced	0.56	0.54	0.28	**0.37**
Unbalanced (random classifier)	0.65	0.31	0.31	**0.31**
Balanced (single predictor)	0.58	0.61	0.57	**0.59**
Balanced (with temporal information)	0.59	0.62	0.58	**0.60**
Balanced (random classifier)	0.5	0.5	0.5	**0.5**

Table S3: Summary of the performance indicators obtained for a logistic regression model to predict the success of start-ups in the open-deal list based on the last and on the mean value of closeness over time. Both unbalanced and balanced cases are considered.

S6.2 Logistic regression: the balanced case

It is well known that many binary classification algorithms suffer if the two classes are unbalanced, i.e. if the number of samples in each class is not similar. A classifier would then systematically try to fit the over-represented class and, as an outcome, the classification would be biased. Consider, e.g., the extreme case where the classifier assigns each sample to the over-represented class. In this extreme situation, the classifier would not be predicting anything, but the classification accuracy would still be very high due to class unbalance. For such a reason most classifiers do not perform well for unbalanced classes, and in unbalanced classification, accuracy can be a misleading metric. This is indeed our case, as in our data set the majority of start-ups do not end up being successful. Here, we show that, when we correct for class unbalancing, then the prediction performance substantially improves. In order to solve the issue

of unbalanced classes, we downsample the over-represented class, so that the successful/non-succesful classification problem has now perfectly balanced ($50\% - 50\%$) classes.

All over this section we use 5-fold crossvalidation. First we have considered that case where we only use the value of the closeness centrality of each start-up in the last month of our observation window, this being closer in spirit to the analysis performed in the main part of the manuscript. Again, the descriptor used is the closeness centrality rescaled ranking. The performance indicators of this logistic regression model are reported in Table S3, while the confusion matrix is shown in Table S4. Results confirm that prediction is indeed possible, and performance indicators are safely superior to random benchmarks.

ACTUAL		**predicted**	
		failure	success
**true**	failure	0.275	0.227
	success	0.193	0.305

RANDOM		**predicted**	
		failure	success
**true**	failure	0.25	0.25
	success	0.25	0.25

Table S4: (Left) Confusion matrix for a logistic regression with a single predictor in the balanced case , using 5-fold cross-validation. (Right) Equivalent confusion matrix expected for a random classifier in the same balanced case.

We have also considered a second logistic regression model with predictors including various statistics of the temporal sequence of closeness centralities in the observation window. We have used the following 9 predictors based on closeness centrality, namely: maximum value, minimum value, slope of a linear interpolation and last value of both the ranking and the rescaled ranking, and number of months in the observation window). The model provides an accuracy of 0.59, sensitivity 0.62 and precision 0.58, indicating that temporal information leads to only a marginal improvement over the previous case.

Finally, we have investigated other logistic regression models by further adding predictors related to other centrality measures. We find that the performance is not boosted, in agreement with the fact that in our case most of the other centrality measures tend to be correlated to the closeness, according to Fig.S9.

3i group	advanced technology ventures	accel partners
andreessen horowitz	atlas venture	atomico
august capital	austin ventures	avalon ventures
azure capital partners	bain capital ventures	balderton capital
battery ventures	benchmark	bessemer venture partners
binary venture partners	canvas venture fund	carmel ventures
charles river ventures	clearstone venture partners	columbus nova
costanoa venture capital	crosslink capital	crunchfund
data collective	digital sky technologies fo	draper fisher jurvetson
elevate ventures	ff venture capital	fidelity ventures
firstmark capital	first round capital	flybridge capital
foundation capital	founders fund	general catalyst partners
genesis partners	golden gate capital	ggv capital
google ventures	granite ventures	greylock partners israel
harris harris group	highland capital partners	idg ventures europe
idg ventures india	idg ventures vietnam	initial capital 2
in q tel	index ventures	innovacom
insight venture partners	intel capital	intellectual ventures
institutional venture partners	inventus capital partners	jerusalem venture partners
jmi equity	kapor capital	kleiner perkins caufield byers
khosla ventures	lightspeed venture partners	lux capital
matrix partners	maveron	mayfield fund
menlo ventures	meritech capital partners	merus capital
morgenthaler ventures	new enterprise associates	norwest venture partners
oak investment partners	oregon angel fund	openview venture partners
polaris partners	radius ventures	redpoint ventures
revolution capital partners	rho ventures	finisterre capital
rre ventures	rothenberg ventures	sante ventures
scale venture partners	scottish investment bank	scottish equity partners
sequoia capital	seventure partners	sevin rosen funds
the social capital partnership	sofinnova partners	spark capital
tenaya capital	third rock ventures	tribeca global investments
union square ventures	us venture partners	vantagepoint capital partners
venrock	wellington partners	

Table S5: List of 101 venture capital funds extracted from `crunchbase.com`. Also available at `https://en.wikipedia.org/wiki/List_of_venture_capital_firms`.

Additional References

30. V. Latora, V. Nicosia and G. Russo, *Complex Networks: Principles, Methods and Applications* (Cambridge University Press, 2017).

31. M. E. Newman, Mixing patterns in networks. *Physical Review E*, **67**(2), 026126 (2003).

32. P. Erdös, A. Rényi, On random graphs I. *Publicationes Mathematicae*, **6**, 290-297 (1959).

33. P. Erdös, A. Hajnal, On chromatic number of graphs and set-systems. *Acta Mathematica Hungarica*, bf 17(1), 61-99 (1966).

34. S. B. Seidman, Network structure and minimum degree. *Social Networks*, **5**(3), 269-287 (1983).

35. S. Wasserman, K. Faust, *Social Network Analysis: Methods and Applications* (Cambridge University Press, Cambridge, 1994).

36. N. Lin 1976. *Foundations of Social Research* (McGraw-Hill, New York, 1976).

37. A. Skrondal, S. Rabe–Hesketh, *Generalized Latent Variable Modeling: Multilevel, Longitudinal, and Structural Equation Models* (Chapman & Hall/CRC Press, Boca Raton FL, 2004).

38. W. A. Thompson, On the treatment of grouped observations in life studies. *Biometrics*, **33**, 463–470 (1977).

39. E. L. Kaplan, P. Meier, Nonparametric estimation from incomplete observations. *Journal of the American Statistical Association*, **53**, 457–481 (1958).

40. D. R. Cox, Regression models and life-tables (with discussion). *Journal of the Royal Statistical Society. Series B*, **34**, 187–220 (1972).
